# Cervical Ectopic Pregnancy with Unsupervised Intake of Medications for Abortion: A Case Report

**DOI:** 10.31729/jnma.8345

**Published:** 2023-11-30

**Authors:** Amod Rayamajhi, Anjana Karki Rayamajhi, Arohi Adhikari, Prapti Basnet, Akhilesh Tiwari

**Affiliations:** 1 Janak Medical and Research Center, Balaju, Kathmandu, Nepal; 2 Department of Obstetrics and Gynaecology, B&B Hospital, Gwarko, Lalitpur, Nepal; 3 Himal Hospital, Gyaneshwor, Kathmandu, Nepal; 4 Department of Radiology B&B Hospital, Gwarko, Lalitpur, Nepal

**Keywords:** *case reports*, *cervical*, *ectopic pregnancy*, *hysterectomy*, *induced abortion*

## Abstract

Cervical ectopic pregnancy is the rarest form of ectopic pregnancy, where implantation occurs in the mucosa of the endocervical canal, below the internal os. Medical abortion is a safe and effective method of termination of early intrauterine pregnancies when carried out under the supervision of trained service providers. Unfortunately, unsupervised misuse of such methods can lead to grave morbidities, especially in ectopic pregnancies. We report a case of a 29-year-old female with cervical ectopic pregnancy, with a history of self-induced medical abortion. Her assumption that her pregnancy had been terminated caused a delay in seeking treatment, which led to complications during management. Ultrasonography revealed features suggestive of cervical ectopic pregnancy and evacuation of the fetus and placenta was done using curettage, during which she had uncontrolled severe vaginal bleeding leading to need of emergency hysterectomy.

## INTRODUCTION

Cervical ectopic pregnancy is the rarest form of ectopic pregnancy, with an incidence of 1 in 8,600 to 1 in 12,400 pregnancies,^1^ where implantation occurs in the mucosa of the endocervical canal below the internal os. It is associated with high morbidity and mortality, especially if there is a delay in diagnosis or treatment, leading to complications like profuse haemorrhage necessitating hysterectomy or even death.^2^ Misoprostol (prostaglandin E1 analogue) and Mifepristone (anti-progesterone) are commonly used for medical termination pregnancy (MTP) of early intrauterine origin.^3^ Unsupervised MTP is associated with higher chances of incomplete abortion, failed abortion, and ruptured ectopic pregnancy.^4^

## CASE REPORT

A 29-year female, (Gravida-5, Para-1, Living-1, Abortion-3) with a previous history of lower segment cesarean section (LSCS) presented to our hospital with painless bleeding per vaginum 10 days back, following 10 weeks of amenorrhoea. She had a positive urine pregnancy test 6 weeks back. There was no history of any contraception used. She gave a history of selfintake of medications for abortion three times in the past, all of which were uneventful. She experienced per-vaginal spotting initially, which made her assume that her pregnancy was non-viable, so she attempted self-induction of medical abortion with misoprostol (Tablet misoprostol 200 mcg/pg two tablets orally and two tablets per vaginally), 4 days prior to presentation at our hospital.

**Figure 1:**
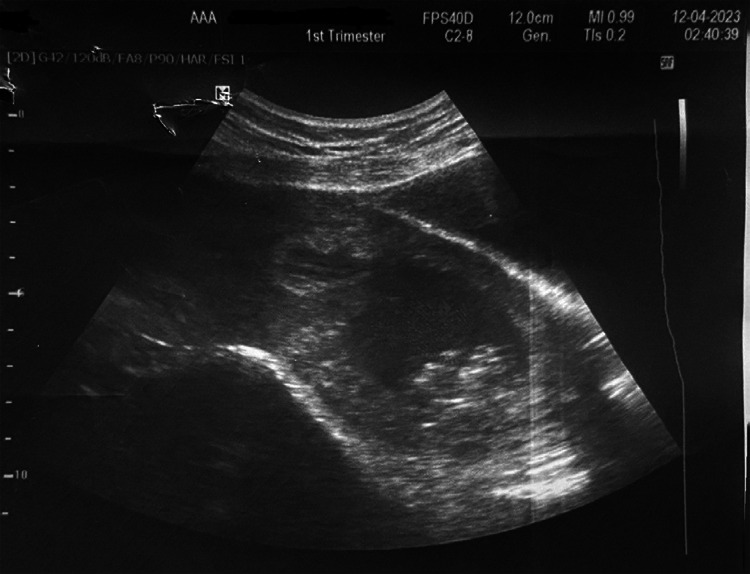
Ultra sonogram showing endometrial thickness.

At presentation, her general condition revealed severe pallor, tachycardia (110-120/min) and blood pressure (BP) of 100/60 mm of Hg. Blood investigations showed haemoglobin 6.4 gm/dL and other parameters within normal limits. Furthermore, per speculum examination showed active vaginal bleeding and bimanual examination revealed a uterine size of 10 weeks, with ballooning of the cervix and product of conception felt at the os. Ultrasonography showed an enlarged uterus (10x5.79x5.73 cm) with an endometrial thickness of 7.4 mm in the upper uterine segment (Figure 1).

**Figure 2:**
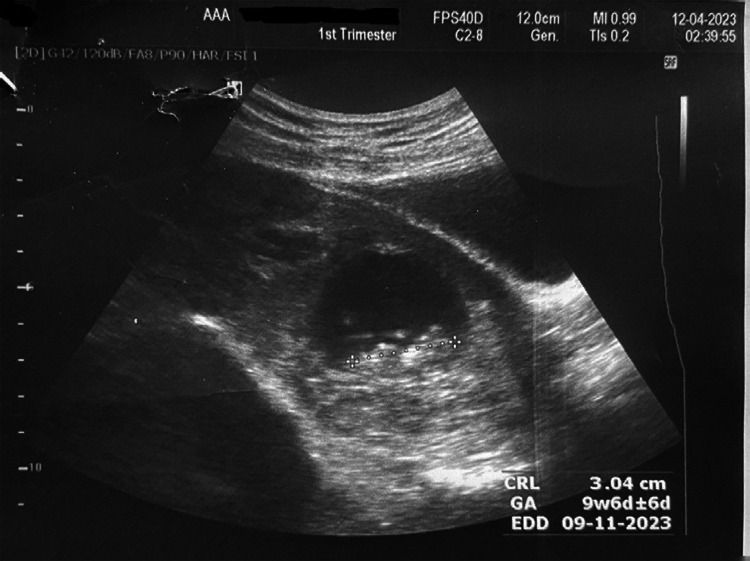
Ultrasonogram showing crown rump length.

**Figure 3:**
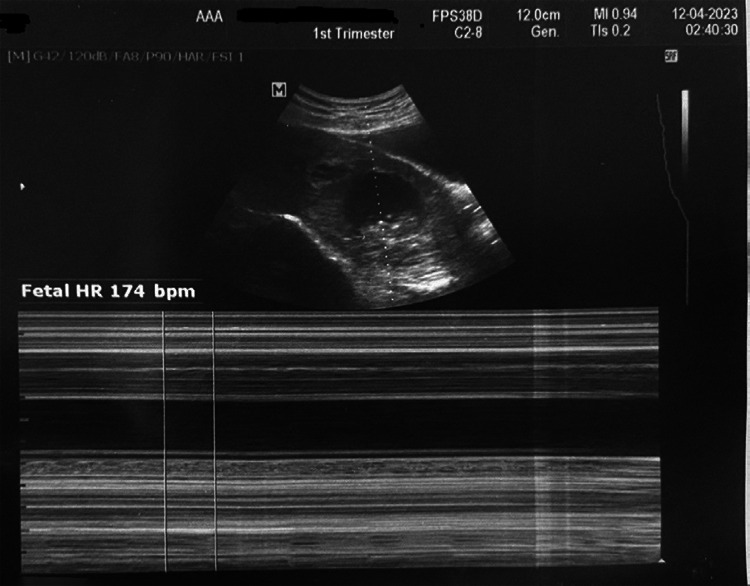
Ultrasonogram showing fetal cardiac activity.

The gestational sac was noted in the cervical canal with a live embryo. The sac contained a fetus with a crown-rump length of 30.4 mm, corresponding to 9 weeks of gestation, with regular cardiac activity of 176 beats per minute (Figures 2 and 3).

Thus, a provisional diagnosis of cervical ectopic pregnancy was made and evacuation of the products of conception was planned. Consent for possible hysterectomy was taken in view of cervical ectopic pregnancy.

**Figure 4:**
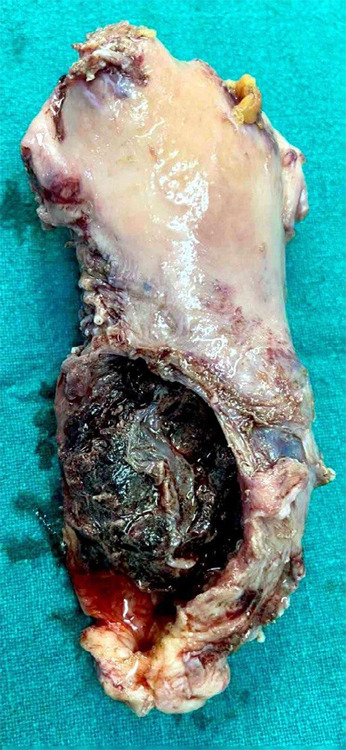
Hysterectomy specimen showing endocervical canal ballooned out with normal body of the uterus.

Evacuation was done, under intravenous anesthesia, removing the fetus and the placenta from the opened os. After evacuation, the bleeding was continuous, so to maintain hemostasis, a Foley catheter (size: 20 Fr) was inflated using 40 ml of sterile water in the cervical canal for tamponade effect. As this was unsuccessful, the condition of the patient gradually deteriorated due to continuous blood loss. In view of her low haemoglobin & continuous bleeding, an emergency total abdominal hysterectomy was performed to save the patient and the specimen was sent for histopathological examination (Figure 4).

Post-operatively, three pints of whole blood, three pints of packed cell and two pints of fresh frozen plasma were transfused. Her haemoglobin then increased to 9.95 gm/dl. On gross examination, a large perforated area was noted on the cervix, 2.5 cm from the os. On cut section, irregular hemorrhagic and friable tissue was noted measuring 5x4.5 cm with some grayish membranous tissue also present.

**Figure 5.**
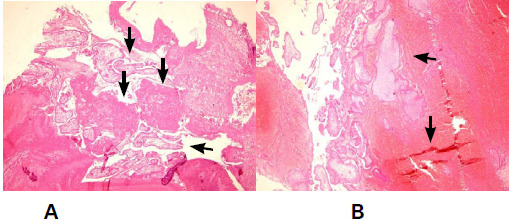
A and B) Chorionic Villi atrophy with trophoblastic tissue with decidualisation and haemorrhage.

A microscopic examination from the perforated area at the cervix showed an extensive area of haemorrhage and decidualization, entrapped within which were a large number of chorionic villi along the trophoblastic tissue. A dense acute inflammatory exudate was noted (Figure 5).

Hence, cervical ectopic pregnancy was diagnosed and the patient was discharged on the fourth postoperative day. On follow-up after one week, the patient was improving clinically with no abnormal bleeding, discharge or any other postoperative complications. The patient was then followed up at regular intervals for a duration of 6 months with no reported complications.

## DISCUSSION

Cervical ectopic pregnancy is a rare but leading cause of maternal morbidity and mortality with a reported incidence of <1% of all pregnancies.^2^ It was first described in 1817 and first named as such in 1860.^5^ It remains a life-threatening condition even with newer modalities and diagnostic tools.^6^ In 1959 five clinically practical criteria were proposed, most of which were fulfilled in our case.^2^

Common predisposing factors are prior caesarean section, prior dilatation and curettage (D&C), and in-vitro fertilization (IVF).^7^ Painless vaginal bleeding is reported by 90% of women with a cervical pregnancy-a third of these have a massive haemorrhage.^1^ There will be a risk of heavy internal bleeding if not treated on time. Because of its rarity, it is often missed out unless they present with a typical triad of clinical findings (amenorrhea, pain abdomen and per vaginal bleeding) and early ultrasonography is performed.

Due to the rarity of the condition and paucity of literature, there are no standard guidelines for the management of cervical pregnancy, but there are some general recommendations.^5^ Medical management with systemic Methotrexate (MTX) is preferred in the first trimester in hemodynamically stable patients. Treatment failure often occurs when the serum human chorionic gonadotropin (hCG) level is >10.000 mIU/mL, the crown-rump length is >10 mm or fetal heartbeat is present.^5^ If cardiac activity is present, intra-amniotic/intra-fetal injection of potassium chloride may be done. When medical treatment fails or is contraindicated, aspiration/curettage or surgical hysteroscopy are possible fertility-sparing alternatives.^5^ However, due to the lack of smooth muscle tissue in the cervical region, there is a predisposition to massive bleeding during conservative surgery.^5^ Hence, if the bleeding is persistent, balloon tamponade by inflating a Foley catheter is attempted. If unsuccessful, further treatment options that preserve fertility include large vessel ligation with cervical cerclage, vaginal ligation of cervical arteries, uterine artery ligation or angiographic embolization of cervical, uterine or anterior iliac arteries. If all conservative and fertility-preserving methods fail, an emergency abdominal hysterectomy is done as a life-saving measure, as in our case.

When a woman opts for abortion in the first trimester, medical abortion (mifepristone & misoprostol) is a safe method for termination of pregnancy when it is consumed under the supervision of a medical practitioner, with a success rate of 92-97%.^8^ WHO guidelines have indicated the requirements of preabortion care for pregnant women needing an abortion, which include: confirmation of the pregnancy, location of the site (intra/extra uterine), estimation of the gestational age and determination of viability.^9^ It also explains that an ultrasound is not mandatory but can be done when available to exclude extra uterine pregnancy and a non-viable one.^8^ The service provider prescribing the medications for abortion must have knowledge about its complications and should ensure access to healthcare facilities for any complications.^9^

Unfortunately, in spite of such extensive guidelines, unsafe and improper use of these drugs by pregnant women under no supervision is surprisingly common. Over-the-counter availability of drugs, leading to unsupervised consumption can lead to incomplete abortion, haemorrhage and missed or delay in the diagnosis of ectopic pregnancies. As in our case, the patient had taken medications for abortion uneventfully, without any supervision/consultation three times, in the past. But this time, as her pregnancy was ectopic in location, not only was it not terminated, but it also caused a delay in presentation leading to severe anemia which led to further complications during her management, ultimately necessitating an emergency hysterectomy.

As cervical ectopic pregnancies are rare and can lead to significant morbidities when presented late, early presentation is key to the success of conservative and fertility-sparing methods. Furthermore, supervised intake of medications for abortion is also of paramount importance, as it prevents their usage in extra-uterine pregnancies and the grave complications that may follow the unsupervised misuse of such drugs. Therefore, all people need awareness regarding timely seeking of health care, especially for obstetric emergencies and for supervised intake of medications for medical abortion.

## References

[1] Cunningham FG, Leveno KJ, Bloom SL, Spong CY, Dashe JS, Hoffman BL (2014). Williams Obstetrics..

[2] Samal SK, Rathod S (2015). Cervical ectopic pregnancy.. J Nat Sci Biol Med..

[3] Parveen S, Rahman MM, Shirin BSN, Rahman M (2019). Fazekas rupture ectopic pregnancy in early gestation due to mifepristone & misoprostol abuse.. Int J Med Res Prof..

[4] Rath S, Mishra S, Tripathy R, Dash S, Panda B (2021). Analysis of complications and management after self-administration of medical termination of pregnancy pills.. Cureus.

[5] Evora F, Hundarova K, Aguas F, Carvalho G (2021). Cervical ectopic pregnancy: a multidisciplinary approach.. Cureus.

[6] Singh S (2013). Diagnosis and management of cervical ectopic pregnancy.. J Hum Reprod Sci..

[7] Sharma A, Ojha R, Mondal S, Chattopadhyay S, Sengupta P (2013). Cervical intramural pregnancy: report of a rare case.. Niger Med J..

[8] Nivedita K, Shanthini F (2015). Is it safe to provide abortion pills over the counter? a study on outcome following self-medication with abortion pills.. J Clin Diagn Res..

[9] World Health Organization. (2015). Safe abortion: technical and policy guidelines for health systems [Internet]..

